# Biotechnological and Immunological Platforms Based on PGL-I Carbohydrate-Like Peptide of *Mycobacterium leprae* for Antibodies Detection Among Leprosy Clinical Forms

**DOI:** 10.3389/fmicb.2020.00429

**Published:** 2020-03-17

**Authors:** Mayara Ingrid Sousa Lima, Fausto Emilio Capparelli, Jaqueline das Dores Dias Oliveira, Patrícia Tiemi Fujimura, Emilly Caroline dos Santos Moraes, Ester Cristina Borges Araujo, Neide Maria Silva, Renata Pereira Alves-Balvedi, Ana Graci Brito-Madurro, Isabela Maria Bernardes Goulart, Luiz Ricardo Goulart

**Affiliations:** ^1^Laboratory of Genetics and Molecular Biology, Department of Biology, Federal University of Maranhão, São Luís, Brazil; ^2^Laboratory of Nanobiotechnology, Institute of Biotechnology, Federal University of Uberlândia, Uberlândia, Brazil; ^3^Laboratory of Biotechnology, Federal University of Tocantins, Palmas, Brazil; ^4^Institute of Biomedical Sciences, Federal University of Uberlândia, Uberlândia, Brazil; ^5^Federal University of Triângulo Mineiro, Iturama, Brazil; ^6^National Reference Center in Sanitary Dermatology and Leprosy, Clinics’ Hospital, School of Medicine, Federal University of Uberlândia, Uberlândia, Brazil; ^7^Department of Medical Microbiology and Immunology, University of California, Davis, Davis, CA, United States

**Keywords:** phenolic glycolipid I, mimotopes, phage display, scFv, ELISA, surface plasmon resonance

## Abstract

Phenolic glycolipid I (PGL-I) is an abundant antigen on the *Mycobacterium leprae* cell wall, commonly used for operational classification of leprosy patients. Our aim was to develop PGL-I mimotopes with similar characteristics and functions of the native antigen. We have used a random peptide *phage display* (*PD*) library for selections against the monoclonal antibody anti-PGL-I. After three selection cycles, six peptides were identified. All sequences were interspersed by a spacer generating a chimeric peptide (PGLI-M3) that was artificially synthesized. The highly reactive peptide was submitted to a reverse *PD* selection with a single-chain Fv (scFv) antibody fragment combinatorial library. The most reactive scFv was then validated by enzyme-linked immunosorbent assay (ELISA) against both native PGL-I and two derived synthetic (NDO and ND-O-HSA). We have further proved the scFv specificity by detecting *M. leprae* bacilli in leprosy lesions through immunohistochemistry. We then described its applicability in ELISA for all clinical forms and household contacts (HC). Afterward, we showed differential binding affinities of PGLI-M3 to sera (anti-PGL-I IgM) from all leprosy clinical forms through surface plasmon resonance (SPR). ELISA IgM detection showed 89.1% sensitivity and 100% specificity, considering all clinical forms. Positivity for anti-PGL-I IgM was twofold higher in both HC and patients with paucibacillary forms in hyperendemic regions than in endemic ones. The SPR immunosensor was able to differentiate clinical forms with 100% accuracy. This is the first time that a PGL-I mimotope has efficiently mimicked the carbohydrate group of the *M. leprae* antigen with successful immunoassay applications and may become a substitute for the native antigen.

## Introduction

The phenolic glycolipid I (PGL-I) is one of the main antigens on the cell wall of *Mycobacterium leprae* and has important roles in the pathogenesis and diagnosis of leprosy (Spencer and Brennan, 2011). The presence of anti-PGL-I antibodies has been mainly correlated with multibacillary forms of the leprosy clinical spectrum and with higher bacilloscopic index (BI) (Lobato et al., 2011), with important applications in household contacts’ monitoring (Frade et al., 2017) and to establish the therapeutic regimens with multidrug therapy (Spencer and Brennan, 2011).

Despite its clinical importance, the extraction and purification of the native PGL-I is restricted to the growth of *M. leprae* in mice and armadillos (Levy and Ji, 2006), due to the natural inability of the pathogen to grow *in vitro* (Youn et al., 2004), leading to a limited availability of the antigen. This problem led researchers to seek for alternatives to native PGL-I using synthetic antigens, such as ND-O-HSA (natural disaccharide with octyl linkage to human serum albumin) and NT-P-HSA (natural trisaccharide with phenolic ring linkage to HSA) (Fujiwara and Izumi, 1987). However, besides their complex synthesis, their reactivities are lower than that presented by the native form (Lobato et al., 2011). Therefore, a new alternative was proposed through the selection of mimetic peptides, but its efficacy for serological diagnosis of leprosy did not work properly (Youn et al., 2004), suggesting that peptides with PGL-I hydrophobic and hydrophilic properties would be difficult to reproduce, especially knowing that the PGL-I antigenicity is conferred by the terminal phenolic disaccharide at the surface (Barnes et al., 2017).

The phage display (PD) technology has been widely used to identify a great number of ligands, including peptides and antibodies (Smith, 1985). So, it is possible to obtain small peptides that mimic specific antigen epitopes (Goulart et al., 2010), or develop biomarkers using Fab (*fragment antigen-binding*) or scFv (*single-chain variable fragment*) antibody fragments (Leow et al., 2014). One of the great advantages of peptides and antibodies obtained by PD is their flexibility to be used in different diagnostic platforms, from conventional enzyme-linked immunosorbent assay (ELISA) (Rojas et al., 2014) to biotechnological platforms, such as those involving immunosensors based on surface plasmon resonance (SPR) (Kim et al., 2015). SPR immunosensors explore the capacity of an antibody to recognize its antigen with different affinities and can be built using an optical signal transduction by a light bean that passes through a prism and reaches a metallic surface (Kretschmann, 1971). These immunosensors represent a great advance to the creation of diagnostic platforms, enabling real time, quantitative, and much more sensitive analysis than the conventional immunoassays (Ngubane et al., 2013).

Here, we present a successful PGL-I mimetic chimeric peptide (mimotope) obtained by PD, which was chemically synthesized with similar immunological properties of the natural antigen. The mimotope was validated by ELISA and SPR, and a reverse engineered antibody against the mimotope demonstrated that the peptide mimics the trisaccharide portion of the antigen. The characterization and diagnostic implications of this novel PGL-I-like peptide are discussed herein.

## Materials and Methods

### Patients Sampling

Patients and household contacts were recruited in the States of Minas Gerais (City of Uberlândia), Brazil, considered endemic region (7.5/100,000 new cases per year). This study was carried out in accordance with the recommendations of the “Guidelines of the National Board on Human Research Ethics” (CONEP) under the approval of the Federal University of Uberlândia (UFU) Research Ethics Committee (CEP 449/10 and CAE 23115003005/2009-36). A written Informed Consent was obtained from each participant.

Leprosy patients were classified according to Ridley and Jopling (1966) in tuberculoid (TT), borderline-tuberculoid (BT), borderline-borderline (BB), borderline-lepromatous (BL) and lepromatous (LL), and submitted to clinical and laboratorial protocols for diagnosis and classification. The number of skin lesions and the bacilloscopic index (BI) of the skin smear were used to determine the operational classification (OC), considering as paucibacillary (PB) patients who had up to five skin lesions and a negative BI, and multibacillary (MB) those with more than five lesions and/or positive BI. Household contacts’ (HC) samples were collected during monitoring, BCG vaccinal scars were recorded and laboratorial analyses were performed.

Sera samples of newborns (*n* = 10) without maternal history of leprosy were used as true negative controls. For specificity tests, visceral leishmaniasis (*n* = 10) and pulmonary tuberculosis (*n* = 10) patients’ sera were used.

### Peptides Selections Through Phage Display

Monoclonal anti-PGL-I antibodies produced in mice (mAb CS-48) were donated by Dr. John Spencer and Dr. Patrick Brennan (Colorado State University), which was used for PD selections. Three cycles of selection with the conformational peptide PD library Ph.D.-C7C^TM^ (New England BioLabs^®^ Inc.), with the initial titer of 1.2 × 10^10^ clones, were performed to select peptide ligands to the anti-PGL-I (500 ng) antibody, which was immobilized into a specific agarose resin (rProtein G Agarose, Invitrogen Life Technologies) that was previously blocked with PBS-BSA 5%, according to the protocol described elsewhere (Barbas, 2001). In each selection cycle, bound phages to the antibody were eluted with glycine buffer (0.2M; pH 2.2), amplified and titrated in *Escherichia coli* ER2738 colony. The selected phages obtained from the non-amplified third cycle were used for extraction and for DNA sequencing using Big Dye Terminator Cycle Sequencing kit together with primer-96 gIII (Biolabs) in a MegaBaceTM 1000 (GE Healthcare) sequencer.

### Bioinformatics and Peptide Design

The *in silico* deduction of the amino acids sequences was conducted through the online tool Expasy Translate Toll (Gasteiger et al., 2003). Modeling of the synthetic peptide was done using the software I-TASSER (Yang et al., 2015) and the molecular structures obtained were visualized and modified using PyMOL 1.5.0.4 (Schrodinger, 2010). After sequence deduction of amino acids from peptides expressed on the bacteriophage surface, the commercial chemical synthesis of the protein motifs with specific design was performed at the Peptide 2.0 (Chantilly, VA, United States). For the design of the synthetic protein, we have created a chimeric molecule with all single selected peptides interspersed with a spacer containing the amino acid sequence PPGGGPP.

### Enzyme-Linked Immunosorbent Assay (ELISA)

High affinity plates (Maxsorp – Nunc^®^) with 96 wells were sensitized with the synthetic peptide (1 μg), or ND-O-HSA (0.05 μg) (Bei Resources^1^) diluted in carbonate/bicarbonate buffer (pH 9.6). The plates were incubated overnight in a cold chamber at 4°C. One washings were performed with 200 μL/well of 1X PBST (Phosphate Buffered Saline with 0.05% Tween-20), and wells sensitized were blocked with PBS-BSA 5% for 1 h at 37°C. Then, sera from all individuals (1:100) were added in triplicate. The plates were incubated for 1 h at 37°C, and after three washings with 1X PBST, 50 μL of human anti-IgM (1:5000) or anti-IgG (1:5000) coupled with peroxidase (Sigma-Aldrich) were used according to the specific experiment and incubated for 1 h at 37°C. After three washings with 1X PBST, reactions were developed by adding 50 μL of OPD (*o*-phenylenediamine dihydrochloride) solution for 5 min (2 mg OPD substrate + 5000 μL citrate buffer + 2 μL H_2_O_2_), and the reaction was then stopped with 20 μL/well of sulfuric acid (H_2_SO_4_ 2N). ELISA readings were performed in a microplate reader (TP-READER, THERMO PLATE) at 492 nm.

For Native PGL-I (Bei Resources^1^) the high affinity plates (Maxsorp – Nunc^®^) were sensitized with this antigen (0.5 μg) diluted in 100% ethanol, until the solvent is completely evaporated. The plate were blocked with PBS-BSA 5% for 1 h and the next steps were similar to the protocol for the synthetic peptide, but the washings were performed only the PBS (Phosphate Buffered Saline) and incubated the room temperature.

Enzyme-linked immunosorbent assay values were converted into an ELISA Index, in which a value of 1.1 was considered a positive threshold. For the ELISA Index (EI) calculation, the absorbance mean value was divided by the cut-off, considering the values greater than 1 as positive. The cut-off value was obtained with absorbance readings of negative controls, and 3 standard deviations were added to the mean (Lobato et al., 2011).

For IgM detection, samples from 90 contacts and 142 patients from Minas Gerais (endemic area) were evaluated with the following distribution of clinical forms: TT (35), BT (32), BB (20), BL (20), and LL (35). For IgG detection, 10 patients from each clinical form and 55 household contacts from the endemic region were evaluated.

### Peptide-Serum Antibody Binding Affinity in the Surface Plasmon Resonance

An electrochemical cell was used for the combined electrochemistry and SPR measurements using an Autolab SPRINGLE system in combination with a PGSTAT 30 Autolab potentiostat. A solid Ag/AgCl electrode was used as a reference, a Pt rod as a counter electrode and the gold surface of the sensor disk (gold covered glass) functioned as the working electrode. For SPR measurements, the Biacore X (GE Healthcare) was also used. In both equipments, the PGLI mimotope (0.03 μg/mL) was immobilized onto a polymeric film Poly(3-hydroxybenzoic acid) 2.5 mM (3-HBA) deposited over the gold electrode surface through Cyclic Voltammetry. The surface was blocked using PBS-BSA 0.25%, and 1:100 diluted sera were added to the sensor disks for evaluation. The standardization of the SPR detection was performed in the Autolab equipment with pools of serum samples from patients, household contacts, and newborns. Afterward, individual tests were performed in Biacore X using samples from five patients from each clinical form.

### Antibody Phage Display

The selection of synthetic peptide PGLI-M3 binding antibodies was done using a scFv library fused to PIII protein (Carneiro et al., 2014) according to the protocol described elsewhere (Barbas, 2001). The combinatory scFv library, obtained from an RNA pool of non-infected individuals, with a 2 × 10^8^ diversity, was amplified using a phage helper properly titrated. For the biopanning, plates were sensitized using 1 μg of the synthetic peptide and blocked with TBS-BSA 3%. The amplified library (7 × 10^9^) was placed into contact with the peptide for 1 h at 37°C. After five successive washes, bound phages were eluted with glycine (0.2M; pH 2.2). The eluded phages were amplified in *E. coli* XL1-Blue for plasmid extraction. Extraction procedure was done using a Miniprep Kit (Qiagen-27106), followed by electroporation in *E. coli* TOP-10 F’. Transformed bacteria were plated and each colony was inoculated in deepwell plates containing SB medium and 2% (v/v) of 2M glucose to obtain soluble scFv. Induction of scFv expression in bacteria was done using 2.5 mM of IPTG (Sigma-I6758). The supernatant containing soluble scFv molecules was later used in ELISA assays against native and synthetic antigens. To further validate the most reactive scFv clone, the antibody was purified in a Nickel affinity column (Histrap HP 5 mL; GE Healthcare) in HPLC (ÄKTA purifier; GE Healthcare) and concentrated by lyophilization (Supplementary Methodology).

### ELISA for Antibody Validation

Firstly, to verify which antibody clones were expressed, a plate was sensitized with the supernatant containing soluble scFv and blocked with PBS-BSA 5%. Detection was performed with the anti-hemagglutinin (anti-HA) coupled with peroxidase (1:2500) as the secondary antibody. The anti-HA was used, because in the structure of the ScFv has one HA epitope used normally in the purification this antibody fragment. Then, to detect the interaction with the target, the ELISA plate was sensitized with 1 μg of the synthetic peptide and blocked with PBS-BSA 5%. The supernatant containing scFv was incubated with the peptide and the same procedures were performed as previously described.

The most reactive scFv was used for other ELISA tests against specific targets, including the native PGL-I, total *M. leprae* sonicate, and total protein extracts of *Leishmania infantum* and *Mycobacterium tuberculosis*. For these assays, the purified scFv was used with a final concentration of 265.8 μg/mL.

### Immunohistochemistry

Histological preparations were obtained from leprosy patients’ skin. Tissue slides embedding was done through freezing using Tissue Tec OCT compound (Sakura) and liquid nitrogen. After sectioning, slides were fixed in 10% formalin. Endogenous phosphatase blocking was made using 5% acetic acid, and 2.5% goat serum was used for unspecific sites. Primary scFv pure antibody (265.8 μg/mL) incubation was done overnight. Secondary and tertiary antibodies, 1:200 anti-His (GE Healthcare) produced in mouse and 1:300 biotinylated anti-mouse (Jackson Lab, code 115.065.003) were used, respectively. Microscopic slides were incubated with the avidin-biotin alkaline-phosphatase complex (1:100, Jackson Lab) and development of the colorimetric reaction was done with fast red Naphthol. A counterstain was done using Harris’ Hematoxylin.

### Ziehl–Neelsen Staining

For *M. leprae* detection, slides were stained in Ziehl–Neelsen’s fenicated fuchsin solution and unstained in acidic alcohol solution (Chloridric Acid 37% and Ethanol 95%). Counterstaining was done using Methylene Blue.

### Statistical Analysis

All statistical analyses were performed using GraphPad prism 6 (GraphPad Software, San Diego, CA, United States). For mean comparisons between groups, a Two-way ANOVA with Bonferroni *post hoc* test was used. Cutoff determination was made using a ROC curve analysis, including sensitivity and specificity parameters, considering patients and their clinical forms, household contacts, endemics controls and newborns.

## Results

**FIGURE 1:**
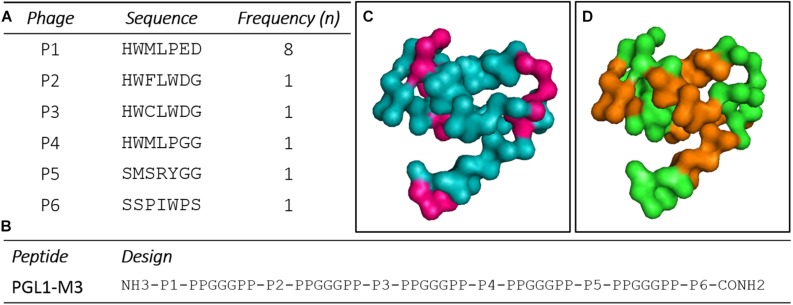
Peptide sequences derived from a random peptide Phage Display (PD) library selected against the CS-48 antibody anti-PGL-I and design of the chimeric protein. **(A)** Peptides sequences and their frequencies. n (number of clones). **(B)** Sequence of the chimeric peptide, PGLI-M3. **(C,D)** Predicted three-dimensional structure of the PGLI-M3 peptide. Meaning of sequence colors: pink (hydrophobic regions), brown (spacers) and green (peptide sequence).

After PD selection, six clones presented high ELISA reactivity to CS-48 (anti-PGLI specific antibody), which were amplified, purified, sequenced, and translated for peptides’ structure characterization (Figure 1A). After structure design, a chimeric peptide PGLI-M3 was developed, which consisted of the six sequences more reactivity (Supplementary Data) interpersed by a spacer, PPGGGPP (Figure 1B). Proline-rich sequences at the spacer ends were incorporated to force protein folding, a strategy used for structural conformation. The predicted structural analysis by bioinformatics indicated that the PGLI-M3 presents hydrophobic regions (Figure 1C), showing similar chemical characteristics to the native PGL-I with a tridimensional conformation (Figure 1D), conferred by proline-rich regions followed by chemically charged amino acids.

**FIGURE 2:**
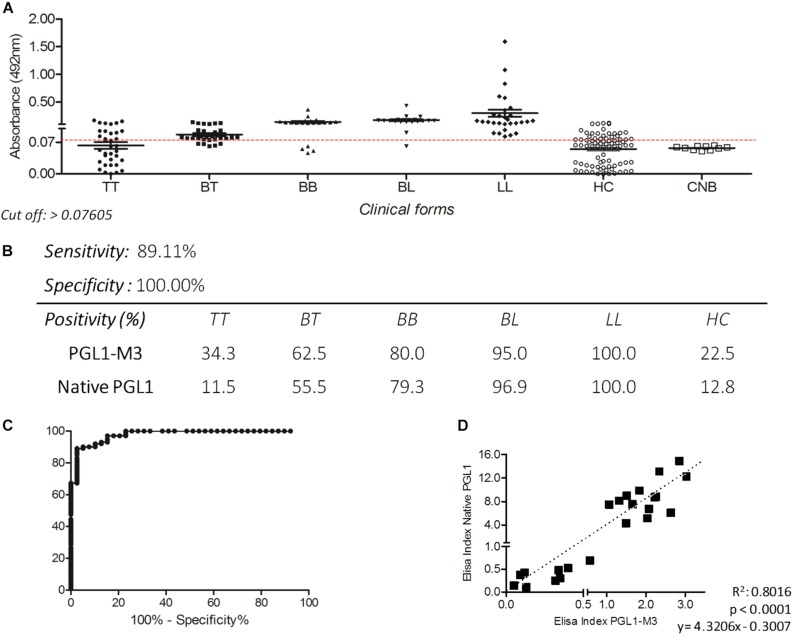
Detection of IgM antibodies by ELISA using the PGLI-M3 antigen in endemic population for leprosy. **(A)** ELISA reactivity of the PGLI-M3 in patients across clinical forms, contacts, and newborns. **(B)** Sensitivity, specificity, and positivity by clinical form. **(C)** ROC curve. **(D)** Linear correlation between ELISA indices of the native PGL-I and the PGLI-M3.

The IgM ELISA with PGLI-M3 in the endemic population achieved a sensitivity of 89.11% and specificity of 100% (Figure 2B), considering an area under the curve (ROC) of 0.9777 (Figure 2C). Positivity was evaluated in all clinical forms, which demonstrated a similar behavior of the native PGL-I, with antibody levels close to 100% in the lepromatous pole (Figures 2A,B). There was a strong positive correlation (*R* = 0.895) between PGLI-M3 and native PGL-I (Figure 2D). For the household contact group, the PGLI-M3 positivity was almost twofold higher (22.5 vs. 12.8%) when compared to the native PGL-I (Figure 2B).

**FIGURE 3:**
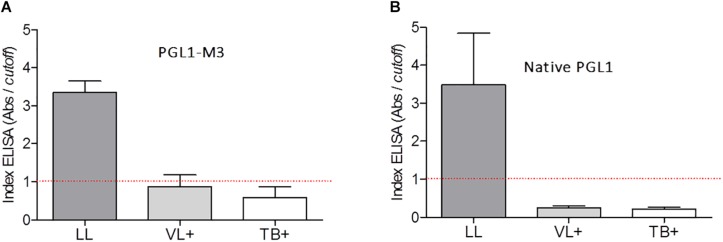
Detection of IgM antibodies anti-PGLI in pool of patients’ samples with lepromatous (LL), visceral leishmaniasis (VL+) and tuberculosis (TB+) using the PGLI-M3 **(A)** and native PGL-I **(B)** antigens. The established cut off is presented in a red line. TT (tuberculoid); BT (borderline tuberculoid); BB (borderline borderline); BL (borderline lepromatous); LL (lepromatous); HC (household contacts); CNB (newborn control).

PGLI-M3 cross-reactivity to the sera from patients with visceral leishmaniasis (VL) and tuberculosis (TB) was compared to the reactivity observed for lepromatous leprosy (LL) patients (Figure 3A). ELISA indices indicated no cross-reactivity, evidenced by values for VL or TB patients lower than the cutoff. The same behavior was also shown for the native PGL-I (Figure 3B).

**FIGURE 4:**
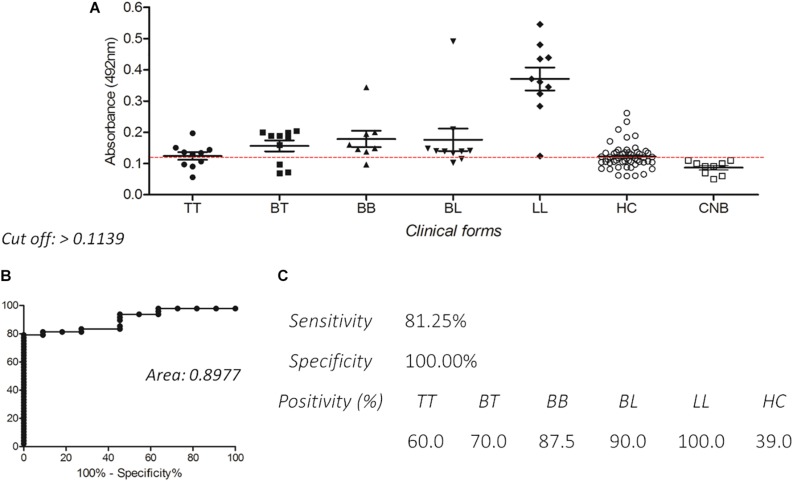
Detection of IgG antibodies by ELISA using PGLI-M3 antigen in hyperendemic population for leprosy. **(A)** ELISA reactivity of the PGLI-M3 in patients across clinical forms, contacts and endemic controls. **(B)** ROC curve. **(C)** Sensitivity, specificity, and positivity by clinical form.

Enzyme-linked immunosorbent assay IgG with PGLI-M3 also presented excellent reactivity in all clinical forms, with very high levels in paucibacillary forms (TT and BT). Positivity was lowest in the TT form (60.0%), and achieved 100% in LL, with increasing levels from the tuberculoid pole toward the lepromatous pole (Figures 4A,C), with a similar behavior observed for IgM. The area under the curve (ROC) of 0.8977 (Figure 4B) reinforces the good sensitivity and specificity of the chimeric peptide, especially in the multibacillary forms (MB). For the contact group, IgG anti-PGLI-M3 positivity was 39.0%.

**FIGURE 5:**
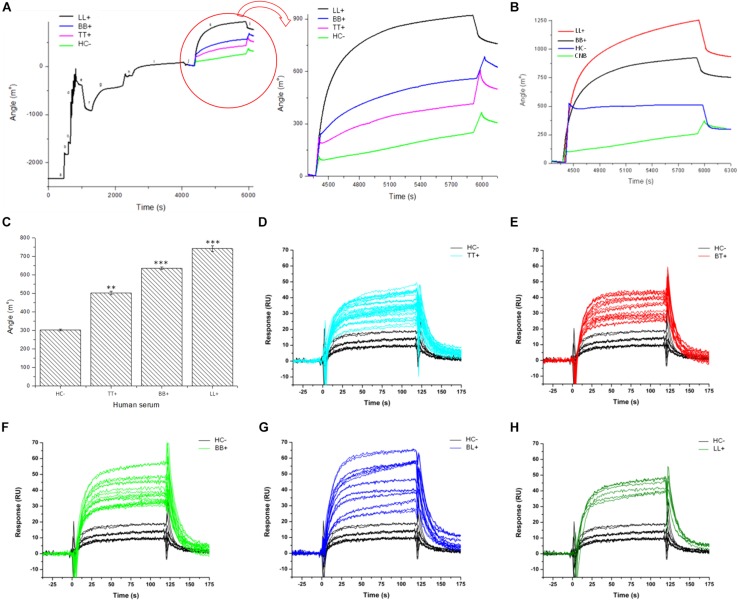
Antigen/antibody binding affinity for leprosy patients measured by Surface Plasmon resonance (SPR). **(A,B)** Sensorgram shows association between PGLI-M3 and antibodies in pools of serum samples from, newborn control (CNB), contacts (HC–), tuberculoid (TT), borderline-borderline (BB) and lepromatous (LL) patients. The graph shows the time (s) and the angular variation (m°). **(C)** Bar graph showing the absolute quantification of the angular variation. **(D–H)** Sensorgrams for the binding affinity for each patient within clinical forms, including the negative control (HC–). **p < 0.05 and ***p < 0.01.

To demonstrate whether the chimeric peptide would present differential binding affinities to serum antibodies of patients, the SPR was first calibrated with pools of sera from three leprosy forms (TT, BB, and LL) and controls. The PGLI-M3 was used to functionalize the sensor disks surface, and significant SPR angle variations were demonstrated for all clinical forms in relation to controls with increasing angle variation across clinical forms, evidenced by lower affinity for TT, moderate affinity for BB and higher affinity for LL. Household contacts did not present significant SPR angle variation (Figures 5A–C). Subsequently, the SPR was then used for individual patients’ analyses, including all clinical forms, and besides being able to distinguish all leprosy clinical forms from controls, it also showed that even paucibacillary forms could be detected, but with lower affinity (Figures 5D–H). This is the first demonstration that a specific biophotonic sensor can distinguish all clinical forms of leprosy from negative controls.

**FIGURE 6:**
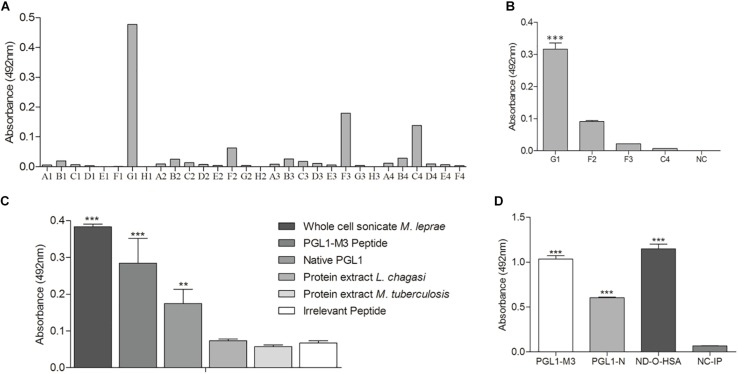
scFv antibody anti-PGLI-M3. **(A)** ELISA detection of scFv antibodies that were expressed in *E. coli* top-10. **(B)** Expressed antibodies that recognized PGLI-M3. **(C,D)** Interaction of anti-PGLI-M3 with different antigens. PGLI-N (native PGL-I); ND-O-HSA (natural disaccharide with octyl linkage conjugated to bovine serum albumin); NC-IP (negative control – irrelevant peptide). **p < 0.005; ***p < 0.001.

To confirm that PGLI-M3 is indeed a peptide that mimic native PGL-I functions, several scFv combinatorial antibodies were generated and the anti-PGLI-M3 antibody was the most reactive one. Out of the 30 bacterial clones that were transformed to express specific scFv antibodies, only the G1 clone was highly significant when tested for binding to the PGLI-M3 peptide (Figures 6A,B). The anti-PGLI-M3 G1 presented the highest reactivity for the whole cell sonicate of *M. leprae*, followed by the PGLI-M3 and then by the native PGL-I, which were significantly different from controls (*p* < 0.001), the proteic extracts of *L. chagasi* and *M. tuberculosis* (Figure 6C), attesting its specificity to PGL-I of *M. leprae*. To further demonstrate its specificity to PGL-I, we have also shown that the scFv recognizes the synthetic molecule ND-O-HSA with even higher reactivity (Figure 6D), a disaccharide form that mimic the trisaccharidic structure of the antigenic determinant of PGL-I, evidencing that the chimeric peptide that originated the new scFv antibody is in fact a peptide that mimic the carbohydrate structure of the *M. leprae* PGL-I.

**FIGURE 7:**
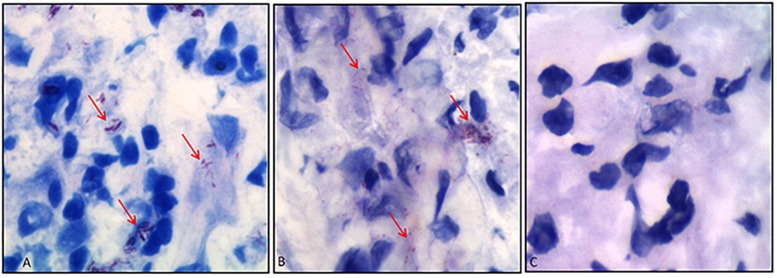
Immunohistochemistry of skin biopsies using anti-PGLI-M3 scFv. **(A)** Identification of *M. leprae* using the Ziehl–Neelsen staining. **(B)** Anti-PGLI-M3 positive labeling. **(C)** Control of the reaction without scFv. The red arrows indicate the bacilli detection.

As a final evidence, we have then tested whether the antibody would bind to *M. leprae* in histopathological analyses. In Figure 7, we have compared Ziehl–Neelsen staining (Figure 7A) with immunohistochemistry (Figure 7B) of skin biopsies from lepromatous patients, in which bacilli presence was confirmed. Results attested that the scFv antibody recognized *M. leprae* (Figure 7B), whereas the analysis in the same tissue without scFv presented no *M. leprae* labeling (Figure 7C).

## Discussion

Currently, the worldwide recognition of PGL-I as a diagnostic tool has led to its intensive use in clinical practice, but its availability is still restricted. In this investigation, we have developed a chimeric peptide that mimic the native PGL-I with important implications in leprosy diagnosis and in monitoring programs of household contacts, and it may become a good substitute for PGL-I. Our novel chimeric peptide was developed due to the production problems of the native molecule that relies on extensive work with armadillo infection and extraction (Levy and Ji, 2006). Alternatives with synthetic molecules have also been used by producing modified carbohydrates that can perform similar functions of the native PGL-I (Lobato et al., 2011), but these methods are also complex, time-consuming, and demand excessive manipulation of reagents (Fujiwara and Izumi, 1987). However, another unusual substitute has emerged, which considered the development of peptides that could mimic PGL-I, but until now the only attempt was unsuccessful and inefficient for leprosy diagnosis (Youn et al., 2004), and this negative result was probably due to the non-specificity of the antibody used, a hypothesis that was not demonstrated.

The application of the surface antigen of *M. leprae*, PGL-I, as serological marker has been well-established (Moura et al., 2008), especially because of its high correlation with high BI and with MB forms of leprosy (Cho et al., 1983), with important role in the operational classification of patients directly affecting the choice of multidrug therapy regimens (Spencer and Brennan, 2011). Furthermore, its use in contacts’ monitoring demonstrated that individuals with high anti-PGL-I titers present a higher risk to develop leprosy and may indicate subclinical infection (Bazan-Furini et al., 2011; Frade et al., 2017).

The diagnostic potential of the chimeric peptide developed in this study can be explained initially by the chemical characteristics of the molecule. The predicted hydrophobic regions on the PGLI-M3 surface may be mimicking the PGL-I lipid structure, while the proline-rich regions of the spacer are forcing its folding, which is fundamental for the molecule to acquire an important conformational structure that joins the peptide motifs that mimic the specific trisaccharide of the native PGL-I. The specific lipid feature is related to the AMA1 mimetic peptide from *Plasmodium falciparum* that also possesses a hydrophobic groove (Alam, 2014). Furthermore, the amino acid sequence has also a saturated ring that favors strong peptide bindings, granting a conformational preference (Lin et al., 2014). Therefore, our chimeric protein structure is functionally like the specific *M. leprae* glycolipid. Another study has also mentioned a peptide that was able to mimic Mannosylated Lipoarabinomannan (ManLAM) from *M. tuberculosis* (Barenholz et al., 2007), but it did not show if the peptide was mimicking the lipid or the carbohydrate domains, although it showed similar reactivity in ELISA ManLAM-based test.

The PGLI-M3 positivity for IgM was detected in MB clinical forms (BB, BL, and LL) follows similar patterns observed for the native PGL-I. Our study observed an average seropositivity of 78% in MB forms, while in the literature the positivity varied from 51.2 to 97.4% for the native PGL-I (Carneiro et al., 2014) and 76.36% for synthetic derivatives with ND-O-HSA ELISA (Lobato et al., 2011). The total positivity including all clinical forms was also similar, for example the lateral flow system (ML-Flow) using NT-P-HSA as antigen presented 97.4% (Bührer-Sékula et al., 2003), which corroborates our findings that showed 92.5% positivity using the PGLI-M3. For the PB group, the positivity has varied from 9 to 31.82% in ELISA assays using the native PGL-I (Lobato et al., 2011; Hungria et al., 2012), while the PGLI-M3 showed a seropositivity close to 50%, which is far above the seropositivity observed for ND-O-HSA ELISA (Lobato et al., 2011) and NT-P-HSA (Bührer-Sékula et al., 2003).

Besides IgM detection, the PGLI-M3 also enabled IgG detection in leprosy patients, with a significant improvement in TT detection (60%). Regarding the IgG subclasses in leprosy, although controversial, it has been previously demonstrated that IgG3 antibody levels were higher in lepromatous than in tuberculoid patients (Beuria et al., 1998). Similarly, it has also been shown that IgG1 was mostly found in LL patients (Dhandayuthapani et al., 1992), whereas IgG2 and IgG4 presented higher levels in TT patients (Hussain et al., 2004). However, it is claimed that IgG2 antibodies are the predominant subclass across the disease spectrum (Beuria et al., 1998). Therefore, it remains to be demonstrated whether our higher detection rates in all clinical spectrum with PGLI-M3 was due to the IgG2 subclass or a mix of them. It has been hypothesized that the mechanism of antibody’s up-regulation is mediated by the IgG2 subclass, in which antibody responses involves increased antigen presentation to CD4+ T cells via activating FcγR+ antigen presenting cells (Getahun et al., 2004), explaining part of detection rates in the tuberculoid pole. In contacts, the IgG detection rate is similar to those observed for other antigens, such as LID-1, ML0405, and ML2331 (Duthie et al., 2011). Our data reinforce the idea that IgG is an important marker of older exposure to *M. leprae*.

Enzyme-linked immunosorbent assay immunoassays with the PGLI-M3 showed excellent efficiency when compared to the native PGL-I, but remarkably this performance was enhanced with the SPR biosensor, which was able to distinguish differential binding affinities of the antigen to serum antibodies from patients in all five clinical forms, which were different from controls. These results represent a great innovation in leprosy diagnostics, because the SPR enabled the detection of low binding affinity of PGLI-M3 to serum antibodies of PB patients, which is not detectable by conventional binding ELISA assays in solution. However, it is not clear whether this indicates distinct physical binding sites for serum antibodies (IgM and IgG) on PGLI-M3, or a complex binding kinetics on a single physical site of the antibodies due to variability in the variable fragment (Fv) of the antibody. Therefore, the differential immunological response among clinical forms is yet to be explained. Interestingly, the binding affinity of PGLI-M3 to serum antibodies measured by SPR was almost 30-fold lower than that measured by a solution binding assay (ELISA), in which the SPR technology was able to detect antibodies with just as few as 0.03 μg of the peptide, while ELISA required 1 μg per reaction. It is important to note that the versatility of the SPR platform has allowed not only the evaluation of peptide ligands to mycobacteria (Ngubane et al., 2013), but also has been used as immunosensors for hepatitis (Villiers et al., 2015) or for detection of salivary proteins (Musso et al., 2015), and also for characterization of binding affinity of human proteins to *M. leprae* glycoproteins (Kim et al., 2015). Another advantage of this platform is the possibility to transform angular variations in absolute values, which present greater application in clinical practice.

To further confirm whether the PGLI-M3 was truly mimetic of the native molecule, we developed a combinatorial antibody (scFv) against the antigen. The produced anti-PGLI-M3 scFv interacted not only with the native PGL-I, but also with the synthetic antigen ND-O-HSA, indicating that the interaction occurs with the carbohydrate structure of PGL-I, which is specific for *M. leprae* (Spencer and Brennan, 2011). This finding was reinforced by immunohistochemistry analyses that demonstrated the direct interaction of the scFv with *M. leprae* in skin biopsies.

Briefly, we have developed through PD a chimeric protein designed with multiple peptide epitopes that mimic the carbohydrate-based antigenic region of the native PGL-I from *M. leprae* with chemical and biological validation. This novel antigen proved to have the same role of PGLI in leprosy diagnostics, especially by using the IgM detection for operational classification. However, we also believe that the IgG detection may also be used for clinical diagnosis, mainly as screening tool. We have also shown through SPR that this novel antigen presents differential binding affinities to serum antibodies of leprosy patients across the clinical spectrum, enabling its detection with greater sensitivity and specificity. Therefore, we present for the first time a fully functional chimeric protein that may replace the native PGL-I for clinical application.

## Data Availability Statement

The raw data supporting the conclusions of this article will be made available by the authors, without undue reservation, to any qualified researcher.

## Ethics Statement

The studies involving human participants were reviewed and approved by the Guidelines of the National Board on Human Research Ethics (CONEP) under the approval of the Federal University of Uberlândia (UFU) Research Ethics Committee (CEP 449/10 and CAE 23115003005/2009-36). The patients/participants provided their written informed consent to participate in this study.

## Author Contributions

ML collected, analyzed and interpreted the data, conceived the research hypothesis, and wrote the manuscript. FC, JD, and PF were collected and analyzed the data. EM interpreted the data and wrote the manuscript. EA and NS collected and analyzed the data of immunohistochemistry. RA-B and AB-M collected and analyzed the data of biosensor. IG collected the clinical data of patients, involved in conceiving and designing the study, conceived the research hypothesis. LG involved in conceiving the study, data analysis and interpretation, as well as reviewing and editing all parts of the final document for publication. All authors read and approved the final manuscript.

## Conflict of Interest

The authors declare that the research was conducted in the absence of any commercial or financial relationships that could be construed as a potential conflict of interest.

The reviewer JS declared a past co-authorship with several of the authors IG and LG to the handling Editor.

## Footnotes

1 https://www.beiresources.org/
