# Supplementary material for: Biotechnological and Immunological Platforms Based on PGL-I Carbohydrate-Like Peptide of Mycobacterium leprae for Antibodies Detection Among Leprosy Clinical Forms
Source: Front Microbiol. 2020 Mar 17;11:429. doi: 10.3389/fmicb.2020.00429 (PMC7092704; doi:10.3389/fmicb.2020.00429)
Supplement: Supplementary file 1 [file Table_1.docx]

**Supplementary Data**

**Suppl. Table 1.** Preliminary ELISA tests with leprosy patients for pre-clinical validation using peptide clones obtained by Phage Display selections against the monoclonal antibody CS-48.

| **Leprosy Patients’ Clinical Forms** | **Number of ELISA Positive Samples / Total Sample**  **(% Positivity)*** | | |
| --- | --- | --- | --- |
|  | **Phage-Clone A3**  **(42%)** | **Native**  **PGL-1** | **Clones’ Pool**  **(6 phage-clones)** |
| **TT** | 0/6 | 0/6 | 4/6 |
| **BT** | 6/9 | 0/9 | 7/9 |
| **BB** | 1/7 | 5/7 | 4/7 |
| **BL** | 3/8 | 8/8 | 5/8 |
| **LL** | 6/8 | 8/8 | 8/8 |
| **Total** | 16/38 (42.1%) | 21/38 (55.2%) | 28/32 (73.7%) |

***P.S:** The use of the most repetitive clone (A3) could detect only 42.1% of positive samples. However, when all phage-fused peptide clones were mixed, the seropositivity was superior of the mixture was greater than that of the native PGL-1 (73.7% x 55.2%). These data were also supported by additional ELISA and SPR analyses with the four different peptides, which were designed using different spacers that allowed differential conformation. These analyses also indicated that the best peptide design was **PGL1-M3**, which was used for all validation assays and for antibody screening. The peptide designs with selected motifs (P1, P2, P3, P4, P5 and P6) and spacers used were as follow:

1. NH3-P1-CGGGSC-P2-CGGGSC-P3-CGGGSC-P4- CGGGSC-P5-CGGGSC-P6- CONH2
2. NH3-P1-GSGSGS-P2-GSGSGS-P3-GSGSGS-P4- GSGSGS-P5-GSGSGS-P6- CONH2
3. **NH3-P1-PPGGGPP-P2-PPGGGPP-P3-PPGGGPP-P4- PPGGGPP-P5-PPGGGPP-P6- CONH2**
4. NH3-P1-GSGSGS-P1-GSGSGS-P1-GSGSGS-P1-GSGSGS-P1-CONH2
