# Supplementary material for: Biotechnological and Immunological Platforms Based on PGL-I Carbohydrate-Like Peptide of Mycobacterium leprae for Antibodies Detection Among Leprosy Clinical Forms
Source: Front Microbiol. 2020 Mar 17;11:429. doi: 10.3389/fmicb.2020.00429 (PMC7092704; doi:10.3389/fmicb.2020.00429)
Supplement: Supplementary file 2 [file Data_Sheet_1.docx]

**Supplementary Methodology**

**Expression and Purification for ScFv (based in Carneiro et al, 2014)**

*Expression of soluble scFvs in E. coli*.

After the selection, plasmidial DNA from XL1-Blue cells was extracted and used to transform electrocompetent non-suppressor TOP10 cells (One ShotTOP10 Electrocomp E. coli, Invitrogen) for soluble expression of scFv molecules. Ninety-six random clones were chosen from plates and inoculated into a 2-ml deep-well plate (NUNC, Roskilde, Germany) in 1 ml of super broth (SB) medium containing 100 mg ml 1 carbenicillin and 40 mM glucose, then followed by incubation for 14 h at 37 1C and shaken at 300 r.p.m. After amplification, 10 ml of cells were transferred to a new deepwell plate containing the same medium and incubated for 4 h at 37 1C and 300 r.p.m., afterwards followed by centrifugation at 3000 g for 10 min. The supernatants were substituted by 1 ml of SB medium containing 100 mg ml 1 carbenicillin and 2.5 mM of isopropyl-b-D-thiogalactoside (IPTG, Sigma, St Louis, MO, USA); after that, the scFv induction was carried out for 18 h at 30 1C and 300 r.p.m. The supernatant containing the scFv was obtained by 3000g centrifugation for 20 min and was directly subjected to ELISA immunoassay and purification.

*Soluble scFv antibody production and purification.*

The scFv clones were transformed in TOP10 *E. coli* non-suppressor strain and then used to inoculate SB medium containing 50 mg ml 1 of carbenicillin and 2% glucose. The culture was grown under agitation (250 r.p.m.) overnight at 37 1C and 2 ml were diluted in 200 ml of supplemented SB medium. The culture was incubated under agitation for 6–8 h at 37 1C (OD600 ¼ 1.0), and centrifuged at 3000 g for 10 min at 4 1C. Bacteria were resuspended in 200 ml supplemented carbenicillin SB medium and induced by 2 mM IPTG at 30 1C overnight. Supernatant containing antibody was obtained after centrifugation at 5000 g, 4 1C, 15 min, after that, His-tagged scFv fragments were purified by immobilized-metal (Ni) affinity chromatography (HisTrap HP, GE Healthcare) by high-performance liquid chromatography (HPLC, Amersham Biosciences, Sunnyvale, CA, USA) according to the manufacturer’s instructions. The positive fractions collected was desalted by a Centriprep column (Millipore, Billerica, MA, USA), lyophilised, resuspended in PBS and then measured at 280 nm for protein.
